# Inferring drug-disease associations from integration of chemical, genomic and phenotype data using network propagation

**DOI:** 10.1186/1755-8794-6-S3-S4

**Published:** 2013-11-11

**Authors:** Yu-Fen Huang, Hsiang-Yuan Yeh, Von-Wun Soo

**Affiliations:** 1 Institute of Information Systems and Applications, National Tsing Hua University, HsinChu, Taiwan; 2 Department of Computer Science, National Tsing Hua University, HsinChu, Taiwan

## Abstract

**Background:**

During the last few years, the knowledge of drug, disease phenotype and protein has been rapidly accumulated and more and more scientists have been drawn the attention to inferring drug-disease associations by computational method. Development of an integrated approach for systematic discovering drug-disease associations by those informational data is an important issue.

**Methods:**

We combine three different networks of drug, genomic and disease phenotype and assign the weights to the edges from available experimental data and knowledge. Given a specific disease, we use our network propagation approach to infer the drug-disease associations.

**Results:**

We apply prostate cancer and colorectal cancer as our test data. We use the manually curated drug-disease associations from comparative toxicogenomics database to be our benchmark. The ranked results show that our proposed method obtains higher specificity and sensitivity and clearly outperforms previous methods. Our result also show that our method with off-targets information gets higher performance than that with only primary drug targets in both test data.

**Conclusions:**

We clearly demonstrate the feasibility and benefits of using network-based analyses of chemical, genomic and phenotype data to reveal drug-disease associations. The potential associations inferred by our method provide new perspectives for toxicogenomics and drug reposition evaluation.

## Background

Disease an intricate phenotype is usually caused by congenital disorder or dysfunctions of abnormal genes which induce multi-factor-driven alterations and disrupt functional modules [1]. Drugs achieve their therapeutic effect by changing downstream processes of their targets which contend with the alterations of those abnormal genes. The previous reports also showed that pharmaceutical company takes approximately 15 years and over $1 billion to develop a novel drug into the market and more than 90% of experimental drugs fail to move beyond the early clinical test stages [2,3]. Because drug discovery is complexity, time-consuming process and there are odds of low therapeutic efficacy and/or unacceptable toxicity [4,5]. With the merits of shorting development time and reducing risk, more and more scientists have been drawn attention to inferring drug-disease associations by computational method. Development of an integrated approach for systematic discovering those associations is necessary.

Several studies investigated some methods to increase the efficacy of drug discovery and they found there are positive and negative relationships between existing drugs and disease phenotypes. The Comparative Toxicogenomics Database (CTD; http://ctd.mdibl.org) is the public database which inferred chemical-disease associations by manually curated chemical-gene interactions, and gene-disease relationships from published literature [6]. Cheng also presented a comprehensive predicted database of chemical -gene-disease associations (PredCTD) by integrating the information from chemical, gene, and disease [7]. Pharmacogenetics and pharmacogenomics knowledge base (PharmGKB) is a repository which contains the relationships between genomics, drug-response and its related phenotype and clinical information [8]. Eichborn developed PROMISCUOUS database which includes network-based resources of protein-protein and protein-drug interactions, side-effects and structural information [9].

The high-throughput microarray technology plays an important role in investigating drug-disease associations by providing a genome-side monitoring of gene expression in the past decade. Some methods aims at restoring the abnormal state to normal state which means the expressions of the transcriptional level induced by drug should reverse those under disease state. On the other hand, if the differential expression profile under drug exposure and disease states is significantly anti-correlated, the drug compounds may have the potential to cure that disease. The Connectivity Map (Cmap) project is one of the most comprehensive and systematic approaches for drug-disease associations [10]. The Cmap provided a reference collection of genome-wide gene expressions profiles among drugs, which were obtained by systematically exposing to few key cell lines [11]. Drug compounds negatively correlated to disease-specific gene signature may be the candidate therapeutic for further investigation. On the other hand, drug compounds positively correlated to gene signature are able to induce the disease phenotype. Li built disease-specific drug-protein associations derived from the Cmap by integrating gene/protein and drug connectivity information based on protein interaction network (PIN) and literature mining from PubMed abstracts [12]. Previous research used the "guilt by association" (GBA) approach, which assumed that when two diseases share similar therapies then the drug treats only one of the two might be also treat another, to predict novel drug-disease associations [13]. With a gold standard set of the drug-disease associations, Gottlieb designed a novel computational method called PREDICT to identify drug-disease associations and also predict new drug indications based on their features including chemical structure, side effects, gene expression profile, and chemical-protein interactome [14]. However, to build an accurate prediction model based on different feature must have the positive and negative data to infer drug-disease associations. There are some technically difficulties to obtain negative data such as non-drug targets due to the lack of value of research. Except learning a classifier to predict the associations between drugs and disease, network- based approach has been widely used to infer the relationships. In genetic and molecular biology, increasing evidences suggested that common functional modules are not affected by an individual gene but usually are organized by a group of interacting genes underlie similar diseases, which point out the therapeutic importance of those modules [15]. Therefore, the other basic hypothesis is that the mechanism of the drug and disease in the pathological processes may share similar functional modules. Daminelli created a drug-target-disease network and mined the bi-cliques where every drug is linked to every target and disease [16]. If the known data form an incomplete bi-clique, the incomplete relations in the bi-cliques to be identified as predicted links between drugs and diseases. Ye integrated known drug target information and proposed a disease-oriented strategy for evaluating the relationships between drugs and a specific disease based on their pathway profile [17].

The huge amount of chemical, genomic and disease phenotype data is rapidly accumulated, but the drug-diseases associations are still not clear. For this purpose, we design a method of inferring drug- protein/gene-disease phenotype relationships with a network propagation model, where genes with similar functional modules are related to not only drugs but also the disease phenotype.

## Methods

**Figure 1:**
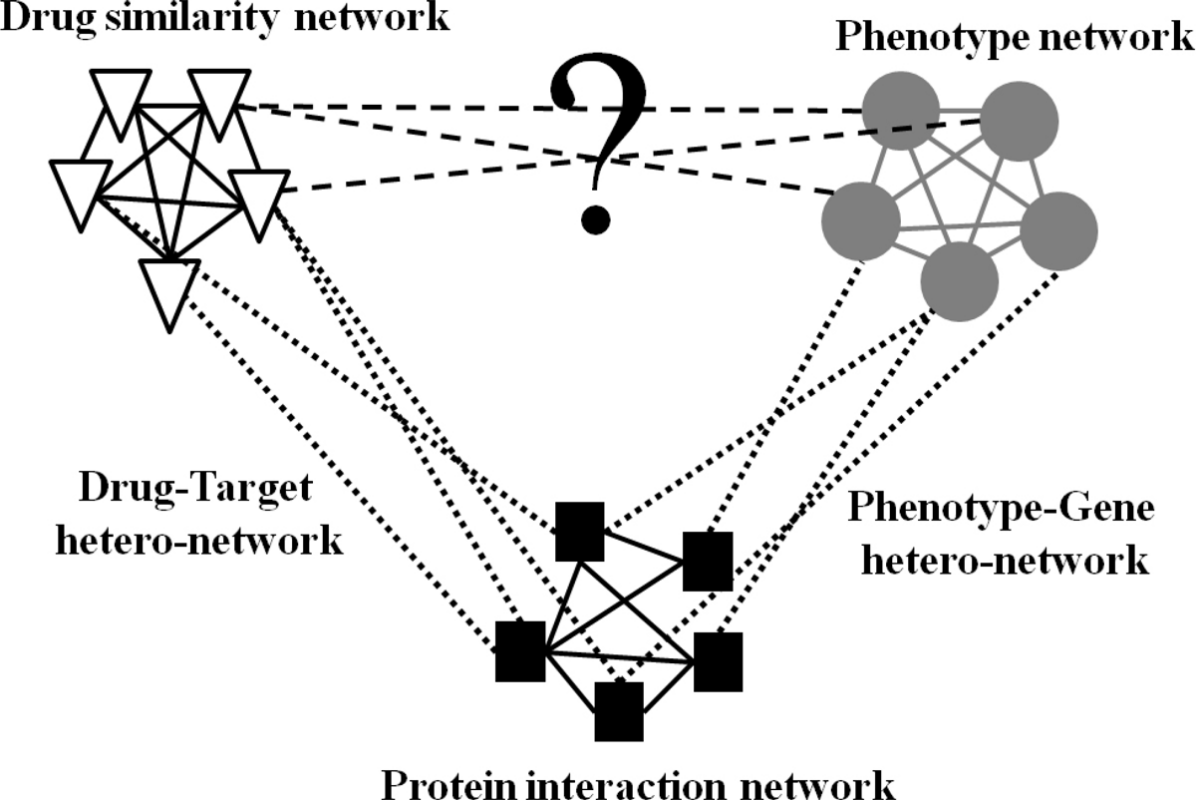
**The idea of our proposed method**.

We demonstrate the integrated network including three heterogeneous networks of the phenotype, drug, protein homo-networks and two hetero-networks capture interactions between two different homo-networks in Figure 1. The homo-network is defined as an undirected graph G_i _= (V_i, _E_i_) where V_i _is the node set and E_i _is the edge set in the homo-network *i*. Connections between two kinds of homo-networks define as hetero-network, where the nodes from different homo-networks are related to each other. The hetero-network is defined as bipartite graph G_ij _= (V_i_∪V_j_, E_ij_). Here, E_ij _represents the set of edges which connect the nodes in different kinds of the homo-networks *i *and *j*.

### Construct phenotype homo-network

A node in a phenotype homo-network as a disease phenotype is extracted from Online Mendelian Inheritance in Man (OMIM) database [18]. We use a scoring schema of phenotypic similarities as edges that quantitatively measures the phenotypic overlap of OMIM records constructed by van Driel [19] using text mining techniques. If the similarity score of two diseases falls in the range [0.6, 1], it means informative similarity which indicates potentially relevant phenotypic similarity. On the other hand, if the similarity score falls within [0, 0.3], it indicates non-informative similarity. Therefore, we apply a logistic function from [20] to convert the phenotypic similarity scores among diseases into a value as close to 1 as possible while the non-informative score into a value as close to 0 possible over all the entries in the phenotype similarity score matrix. The symmetric similarity matrix *W_p_(p_i_,p_j_) *in phenotype homo-network denotes the phenotypic similarity score between phenotypes *p_i _*and *p_j_*.

### Construct drug homo-network using chemical similarity

We extract the FDA-approved drugs and their canonical simplified molecular input line entry specification (SMILES) from DrugBank database [21][22]. We calculate the hashed fingerprints using Chemical Development Kit (CDK) [23]. The chemical similarities are calculated by two hashed fingerprints using Tanimoto coefficient [24]. It calculates the size of the common substructures over the union between two fingerprints of the drugs which is defined as *sim(x, x')*=|*x*∩*x'*|/|*x*∪*x'*| between two chemical structures of drug *x *and *x'*. The symmetric chemical structures similarity matrix as the edge weight in drug homo-network is denoted as *W_d _*and each value falls in the range between zero (no bits in common) to unity (all bits the same)

### Construct protein interaction homo-network using gene expression data

Protein-protein interactions provided an opportunity on the discovery of relationships among proteins in the mechanism of the drugs and human disease phenotype. However, the PIN has its disadvantages: first, the information in current PIN databases is partly complementary and the combination of the multiple databases could improve the knowledge of the protein interactions [25]. Second, PIN in those databases only provides the functional relations among the products of the genes and do not provide information about the conditions under which the interactions occur. Genome-wide expression profile can help us to extract the changes of the genes which are involved in the activity of a given disease or drug compound by comparing multiple case-control data sets. The co-expressed genes can reveal specific linkages which are more likely to function together, and we apply Pearson correlation coefficient for every pair-wise relation among genes. The positive correlation indicates an increasing linear relationship and negative correlation indicates a decreasing linear relationship. On the other hand, the correlation approaches to zero shows there would be little or no association. By visualizing gene expression over-expressed and down-expressed functional modules, we take the absolute value of correlation value to capture inhibitory activity (negative correlation) as well as activation activity (positive correlation). We define the weight function as the product of the absolute value of correlation and the sum of the absolute value of differential expression changes between two corresponding genes in control and case samples. The symmetric weighted matrix between all interactions among all gene pairs is denoted as *W_g _*and the higher weight denotes the stronger correlation or larger differential expression exchanges.

$$W_{g}\left(g_{i},g_{j}\right)=\left|R_{g_{i}g_{j}}^{d}\right|\times \left(\left|E_{g_{i}}^{d}-E_{g_{i}}^{n}\right|+\left|E_{g_{j}}^{d}-E_{g_{j}}^{n}\right|\right)$$

where *W_g_(g_i_,g_j_) *denotes the weight function from gene *g_i _*to gene *g_j_*. $\left|R_{g_{i}g_{j}}^{d}\right|$ denotes the absolute value of Pearson correlation coefficient of the interaction between gene *g_i _*and *g_j _*from case data. $E_{g_{i}}^{d}$ and $E_{g_{i}}^{n}$ are the average gene expression values of gene *g_i _*in case sample and control sample.

### Integrated disease, protein interactions and chemical homo-networks

The gene-phenotype hetero-network shows the relationships between disease phenotype and disease-associated genes extracted from OMIM database [18]. The drug-protein hetero-network denotes the drug and its targets which is obtained from DrugBank database [21]. The asymmetric matrices *W_pg_, W_dg _*represent the adjacency matrices of link structures from phenotype-gene relationships and drug-target protein interactions, respectively. If drug *d*_*i *_has a target *g_j_*, then *W_dg_(d_i_, g_j_) *= 1, otherwise *W_dg_(d_i_, g_j_) = 0*. When a drug target or disease-associated gene has no link with other proteins in PIN, we set the probability of connection to any other protein as 1/(*n*-1), where *n *is the total number of proteins in PIN. Since n is usually very large, so the probability will be very small. The reason that we use small probability instead of zero probability is to prevent a node in the network becoming a "sink node" in PIN and allows the probability to be propagated through the node.

### Network propagation in the integrated network

We identify the inferring drug-disease associations problem as probability propagation over a network which simulates a random walker stochastically move on query phenotype to its immediate neighbors in heterogeneous network [26,27]. We adopt the idea from [28] which developed a label propagation algorithm for an integrated network.

In order to balancing the influence on the nodes which they are connected in the network, we normalize all similarity matrices *W *to be a transition matrix *S *by a diagonal matrix *D_r_(i,i) *which indicates the sum of row *i *while another diagonal matrix *D_c_(j,j) *indicates the sum of column *j *in the similarity matrix, respectively. If the similarity matrix of a homo-network is symmetric, the diagonal matrix *D_r_(i,i) *is equal to *D_c_(j,j)*.

$$S\left(i,j\right)=W\left(i,j\right)/\sqrt{D_{r}\left(i,i\right)D_{c}\left(j,j\right)}$$

where *i *denotes the node in a homo-network and *j *denotes the node in the other homo-network.

Given transition matrix *S*, diffusion parameter α and the vector *p^0 ^*with the initial probability distribution over nodes, the probability transition process in network propagation method on single network within *t *steps is defined as:

$$p^{t}=\left(1-\alpha \right)p^{0}+\alpha Sp^{t-1}$$

The first term denotes the random walker can "restarts" to the initial probability distribution among the nodes with the diffusion parameter 1-*α*. The second term denotes an iterative walk to reach the further nodes in the network based on the transition matrix *S *and a diffusion parameter α. The random walker will be trapped at initial nodes if α is zero. Let *P^t ^*be a probability distribution where a node in the network holds the probability of finding itself in the iterative random walker process up to the step *t*. After certain steps, the probabilities will reach a steady state which the difference between *P^t ^*and *P^t-1 ^*measured by *L2 *norm falls below a very small value such as 10^-9^.

We extend the network propagation on a single network to our integrated network. The nodes receive the probabilities from other nodes in the same homo-network, and also can get the probabilities from nodes in other homo-networks through hetero-networks [29]. Therefore, the initial probability would be replaced by adding the additional information from its immediate neighbors through hetero-networks [30]. The new initial probability vector *p*_i_^0 ^in each homogeneous network *i *is proposed by a weighted sum which is formulated as follows:

$$p_{i}^{0}=a_{i}p_{i}^{0}+b_{ij}\underset{i\neq j}{\sum }S_{ij}p_{j}^{0}$$

Where *a_i _*and *b_ij _*denote the weights in homo-network *i *and those between two homo-networks *i *and *j*, respectively. *S_ij _*denotes the normalized transition matrix of the hetero-network. Then, we should keep the probability of the node be equal to one and we must further ensure the parameters a_i _and b_ij _to satisfy:

$$a_{i}+\underset{i\neq j}{\sum }b_{ij}=1$$

If homo-network *i *connect to other *k *homo-networks, we adopt the weight of b_ij _the same as diffusion parameter *α_i _*to the immediate neighbors in the other homo-networks_. _We calculate *a_i _*+ *kα_i _*= 1 and obtain the weight *a_i _*= 1 - *kα_i_*. Finally, we further elaborate the network propagation method on a homo-network *i *into:

$$\begin{array}{c} p_{i}^{t}=\left(1-\alpha_{i}\right)\left[\left(1-k\alpha_{i}\right)p_{i}^{0}+\alpha_{i}\underset{i\neq j}{\sum }S_{ij}p_{j}^{0}\right]\\ \;+\alpha_{i}S_{i}p_{i}^{t-1} \end{array}$$

Thus, network propagation is calculated with an enriched initialization from the other homo-networks through hetero-subnetworks and the proof of convergence is in [31].

Given a query phenotype, we first set the initial probability distribution over nodes where the probabilities to the query disease nodes set to one and other nodes in the other homo-networks to zero. Second, we apply our network propagation method iteratively until the probability converges on each homo-network. Finally, we use the coverged probabilities of the nodes in homo-networks as initail probability distribution and then repeated the network propagation processes until all homo-networks converge to a final probability distribution.

### Evaluation of association specificity between drug and disease

In our method, we apply chemical similarity, gene expression, and phenotype similarity data and the transition in the network propagation processes may skew the visitation frequencies towards those supplied data values. Since the frequencies of node visitations may also be highly biased by the linkages in the network topology, the probabilities of the nodes may directly reflect the relative centralities in the network based on the local network connectivity [32]. In order to control the topological biases in PIN, we calculate the reference visitation frequencies without taking the gene expression profile of the specific disease phenotype into consideration. Therefore, we set all the edge weights among genes to one in PIN and get the reference probabilities distribution *P_i_^ref ^*over nodes in each homo-network *i *using our method. Then, we evaluate the specificity of the probability of a node using Z-score as the final normalized score distribution which reflects the relevance nodes related to the query phenotype.

$$Z_{i}\left(v\right)=\frac{P_{i}\left(v\right)-avg\left(P_{i}^{ref}\right)}{std\left(P_{i}^{ref}\right)}$$

Here, *P_i_(v) *denote the probability of node *v *in homo-network *i *using genomic data calculated by our method. The functions *avg *and *std *denote the average and standard deviation for the set of reference probabilities *P_i_^ref ^*in homo-network *i *respectively.

## Results

### Gene expression profile

We adopt microarray data taken from [33] that consists of 62 primary prostate tumors and 41 normal tissues from Stanford Microarray Database (SMD) [34]. We use genome-wide gene expression profiles from tissue samples of 18 healthy normal controls and 27 patients with colorectal cancer evaluated by HG-U133 Plus 2.0 platform microarrays (Affymetrix, Santa Clara) through from Gene Expression Omnibus (GEO) database (GSE4183 and GSE4107) [35,36].

### Protein interaction network

We successfully obtained 137,037 interactions among 13,388 genes by integrating five protein interaction network databases (HPRD, BIND, IntAct, MINT, and OPHID) and by mapping the UniProt protein ID to the human Entrez gene ID, erase the duplicated interaction pairs.

### Phenotype network and Phenotype-genotype hetero-network

The OMIM database constructed the catalogue of genetic diseases in human and provides the phenotype-genotype association for 14,433 genes and 5,080 diseases [18]. The gene-phenotype hetero-network contains 275 disease phenotypes and 649 genes from 877 relations while mapping the genes in microarray data and PIN.

### Drug network and drug-target hetero-network

We collect 1,571 FDA-approved drugs and 1,410 of them with available SMILES data in DrugBank database [21]. There are 4,456 relations between 1,215 drugs and 1,141 targets to be the drug-target hetero-network.

### Benchmark of drug-disease associations

We extract 53 and 106 known associations between drug, prostate cancer and colorectal cancer extracted from CTD database [6] in May 2013 as our benchmark.

### The performance of our method

**Figure 2:**
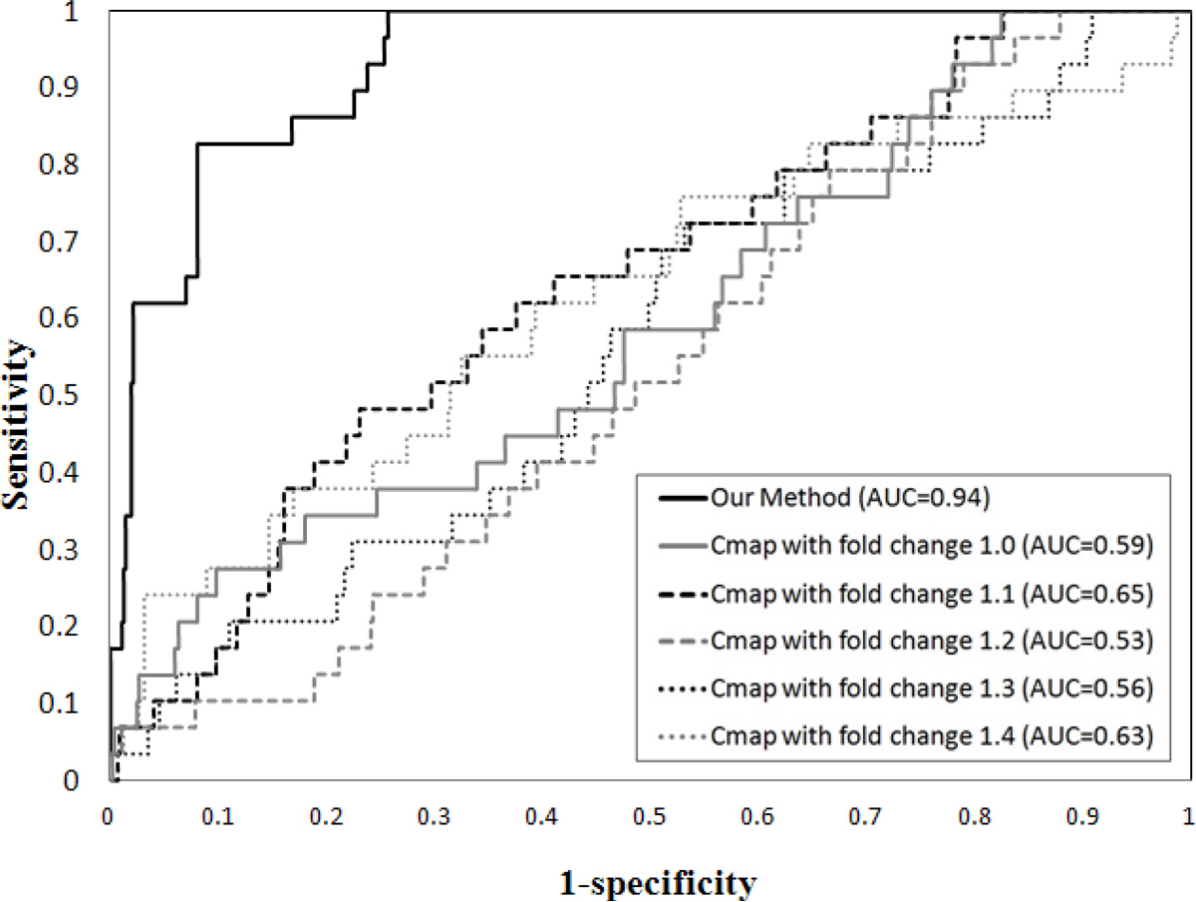
**ROC curve among Cmap and our method in prostate cancer**.

**Figure 3:**
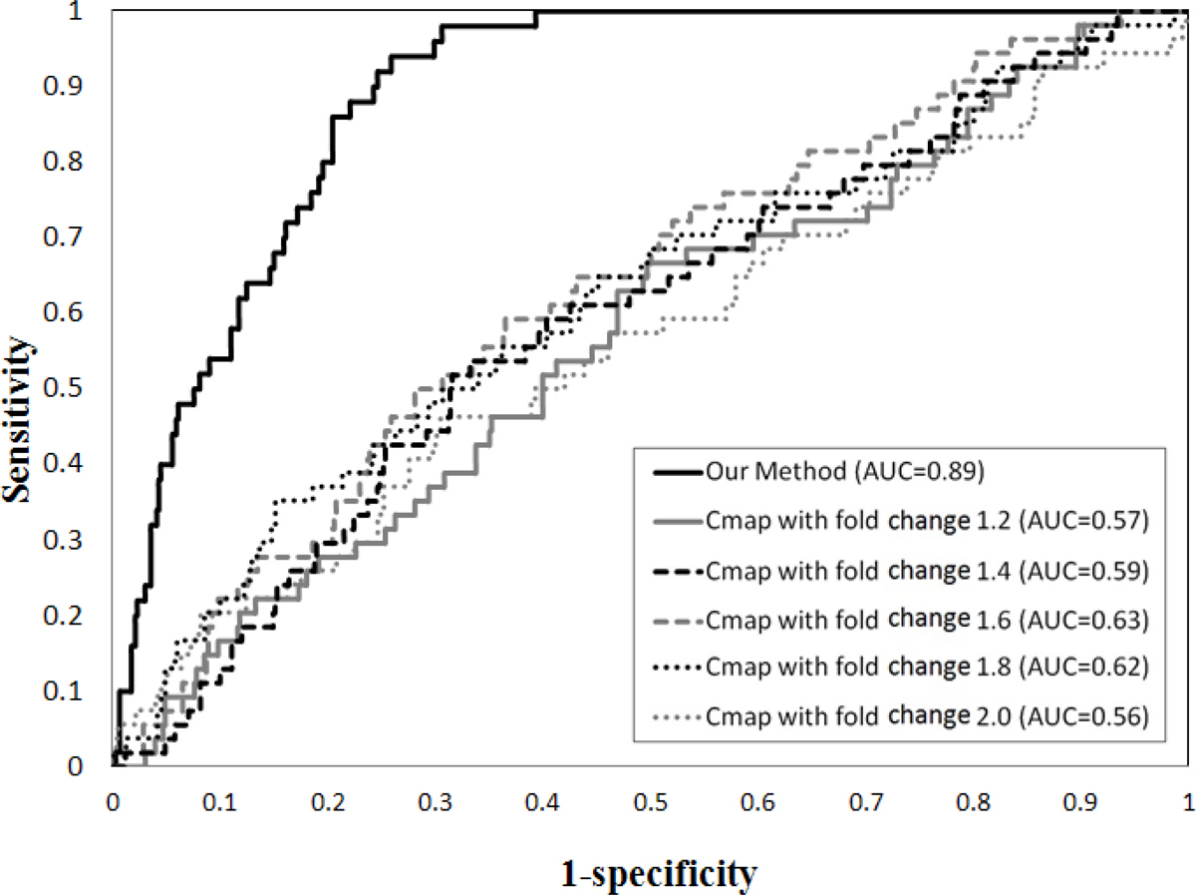
**ROC curve among Cmap and our method in colorectal cancer**.

We compare our method with the previous state-of-the-art Cmap project to evaluate the performance [10]. We first prepare the ranked lists of over-expressed genes and down-expressed genes in both prostate and colorectal cancers which are conducted with affymetrix HG-U133A platform for Cmap project. The number of over-expressed and under-expressed gene signature in prostate cancer and colorectal cancer microarray data with different fold changes are shown in Table 1. Given the disease-specific gene signature as input query, the drug compounds ranked lists obtained from Cmap which are scored based on how well they are correlated with the input query. The score of 1 represents the input query perfect matches the changes among drug expression profiles in the database, -1 represents perfect anti-correlation between them, and 0 represents a null match. Both negative and positive enrichment belong to the drug-disease associations. Therefore, we take the absolute values of scores and re-rank them and higher score represents the stronger drug-disease associations. Due to Cmap project is derived from the expression profile of a drug compound to the isolated cell lines, multiple instances may correspond to same drug and even with same dose in the obtained results. We calculate the maximum score of multiple instances that correspond to the same drug as the associated score of a specific drug for dealing with such cases. In prostate and colorectal cancer data, we use 601 drug compounds which are overlapped between DrugBank and Cmap results and the drug rank list have shown in the previous works [37]. We compute observed the area under the receiver operator characteristic (ROC) curve (AUC) for analyzing the quality of performance in Figures 2 and 3, respectively. The Cmap method obtains much lower AUC of 0.59 ± 0.04 and 0.59 ± 0.03 under the gene signature with different fold changes while our method obtains 0.94 and 0.89 in prostate cancer and colorectal cancer respectively. Previous study showed that only depending on the drug response expression profile is incapable to acquire the drug-disease associations accurately due to the profiles generated under different conditions [38]. There are several problems that limit the performance in Cmap project: First, the set of differentially expressed genes that constitute disease signatures or drug signatures were chosen empirically that cannot guarantee the biological relevance of the selected signatures. A bad selection of signatures may tend to capture similarities in the experimental settings rather than revealing the underlying mechanisms. Second, only the overlap among genes between disease state and drug treatments are not quantified as the overall effect of a drug and the values of differential expression are also not taken into account in Cmap project. Therefore, the feasibility and benefits of using network-based analyses of chemical, genomic and phenotype data provides a good chance to reveal drug-disease associations.

**Table 1:** The number of gene signature with different fold changes in prostate cancer and colorectal cancer

Cancer	Prostate cancer					Colorectal cancer				
**Fold change**	**1.0**	**1.1**	**1.2**	**1.3**	**1.4**	**1.2**	**1.4**	**1.6**	**1.8**	**2**
**# over-expressed genes**	89	60	45	31	27	539	308	175	106	68
**# down-expressed genes**	232	156	115	86	58	72	42	27	12	5

### The AUC with varying diffusion parameters

**Figure 4:**
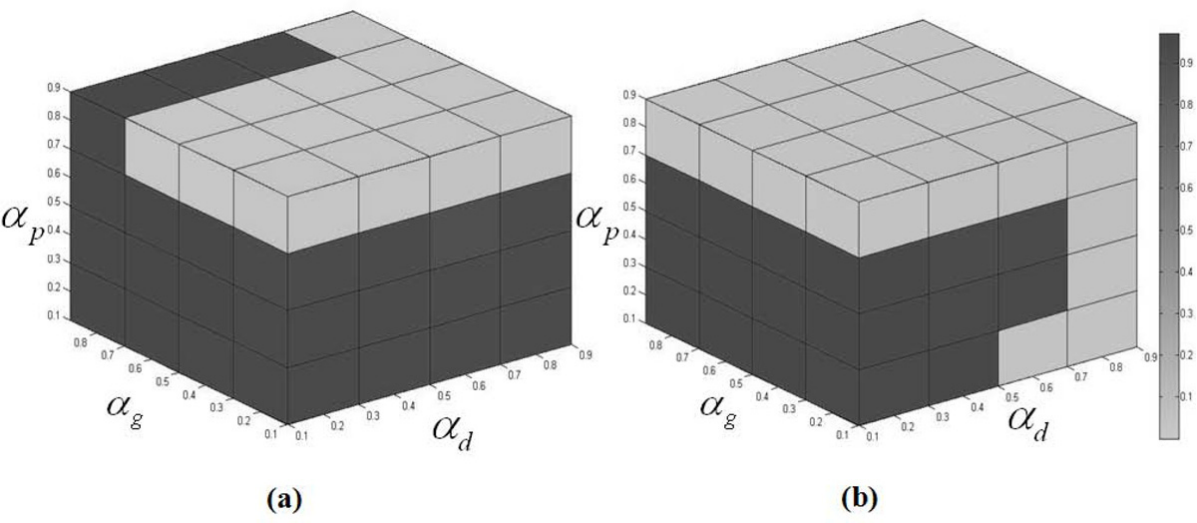
**AUC comparison among the different combinations of the diffusion parameters in (a) prostate cancer and (b) colorectal cancer**.

We investigate the systematic effect of the different diffusion parameters among networks in our approach. We apply the diffusion parameters α_i _{0.1, 0.3, 0.5, 0.7, 0.9} used in the experiments and 125 combinations of the parameters are tested. The ranking performances of our method with different combinations are measured by AUC values in Figure 4. The results show that the diffusion parameters set in drug, gene, and phenotype homo-network α_d, _α_g, _and α_p _as 0.1, 0.7 and 0.3 can get a higher AUC in both prostate and colorectal cancer. A higher similarity score between the chemical structures of a pair of drugs may sometimes have different targets to affect different functional modules in PIN. The drugs with similar chemical structures without binding to similar enzymes would reduce the predictive accuracy for drug similarity [39]. On the other hand, the bit-comparison method of chemical structures using Tanimoto coefficient also has its limitations [40]: First, this kind of method does not include biochemical information at the atomic level of the representation. Second, sometimes it may yield low similarity values and it has an inherent bias related to the size of the molecules that are being sought. Furthermore, many physiological effects cannot be predicted accurately by chemical structure properties alone without more detailed information of metabolic and pharmacokinetic transformations of drugs [41]. Those may be the reason that why the diffusion parameter value in the drug network should be relatively smaller in our experiment. PIN tends to have much higher diffusion parameters to get higher AUC and it is reasonable to infer drug-disease associations not only by targeting the specific proteins directly but also by modulating the pathways involved in the pathological process [42].

### The performance of our method with different data source

**Figure 5:**
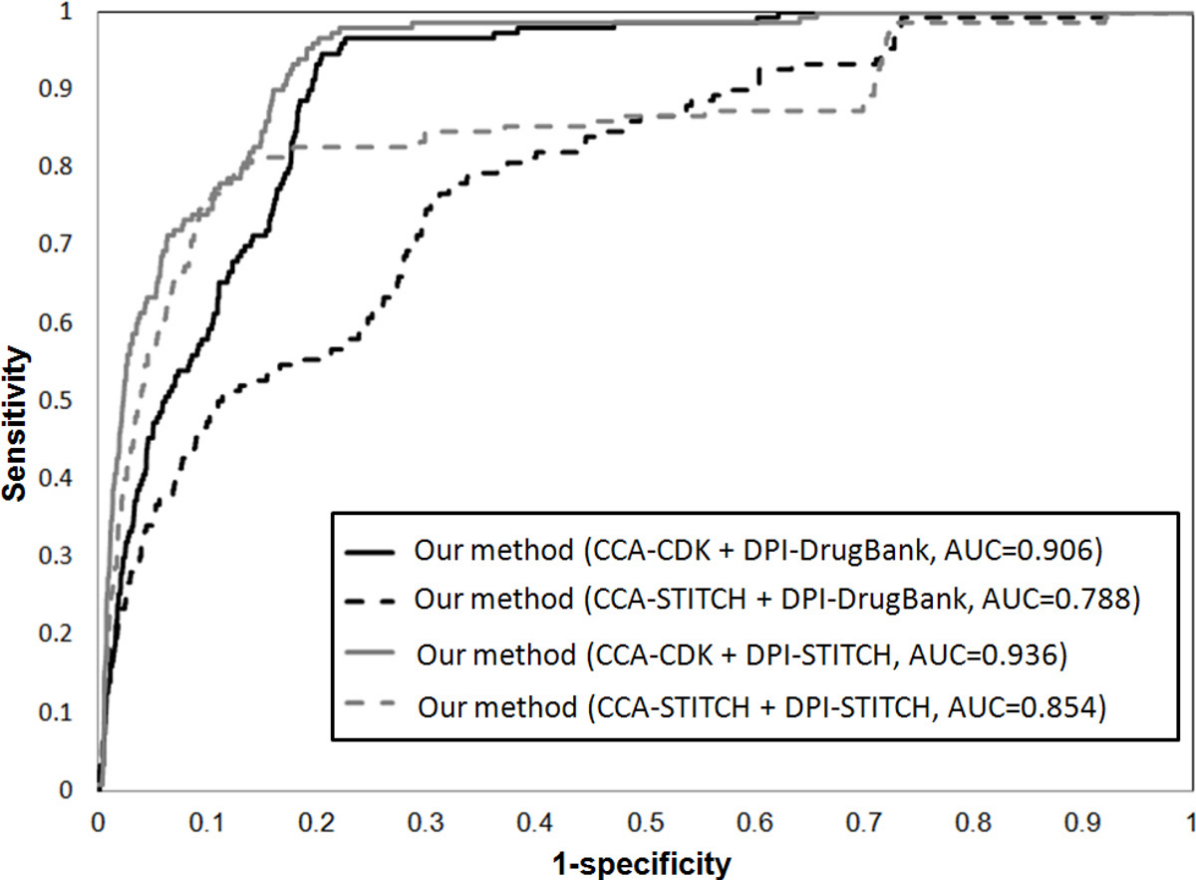
**ROC curve of our method with different data source supported in prostate cancer**.

**Figure 6:**
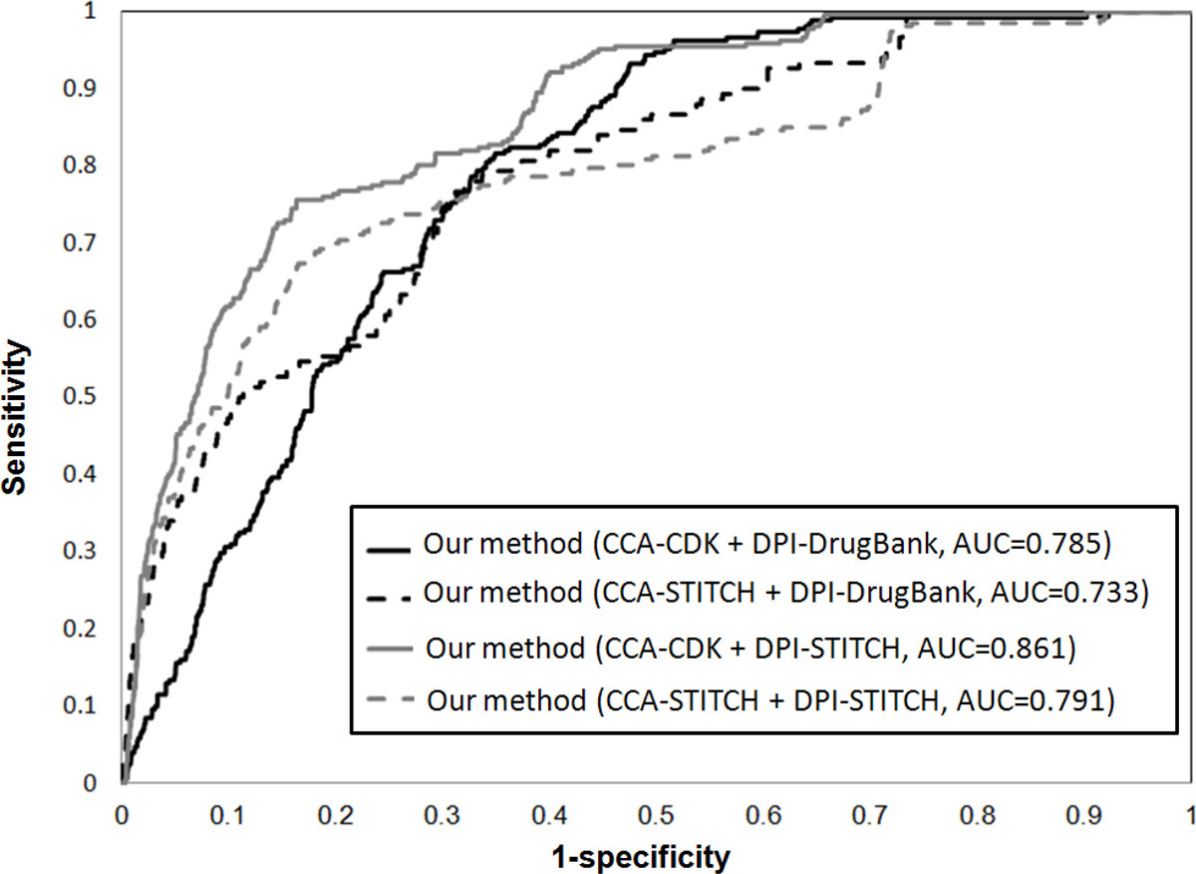
**ROC curve of our method with different data source supported in colorectal cancer**.

The drug is designed to target on the primary targets, but it also may interact with unexpected proteins (called off-targets) to trigger the associated biological processes and pathways. It may display undesired off-target toxicity or drug reposition for the disease phenotype. Therefore, we aim to add additional drug targets information to better understanding of dynamic processes under drug exposure. The database STITCH contains the relations between chemicals as chemical-chemical associations (CCA), and chemicals and their interacted proteins as drug-protein interactions (DPI) which are all curated by the evidence derived from experiments, publicly databases and the literature extraction [43]. However, the textual co-occurrence from text mining does not necessarily indicate meaningful relationships [44]. Therefore, we take 29,275 chemical-chemical interactions and 45,567 chemical-protein pairs from STITCH excluded the relations extracted from the text mining method. In the rank lists of 1,410 drugs, our method with the chemical structures similarity from CDK in drug homo-network and the chemical-protein interactions from STITCH in drug-protein hetero-network obtain highest AUC of 0.936 and 0.861 in prostate cancer and colorectal cancer dataset in Figure 5 and 6, respectively. It shows that our method with more potential off-targets from STITCH gets higher AUC than that with only primary drug targets from DrugBank in both prostate cancer and colorectal cancer data. Due to only a limited number of chemical-chemical associations have been identified in STITCH [45], its performance is worse than the associations constructed by chemical structures similarity from CDK in drug homo-network.

### Case study: prostate cancer

#### Potential drug and prostate cancer relations

We run our method with diffusion parameters α_d, _α_g, _and α_p _as 0.1, 0.7 and 0.3 in drug, gene/protein, and phenotype homo-networks and display 26 drug-prostate cancer associations by evaluating the one-tailed test at 0.01 level of significance with Z-score > 2.33 in Table 2. Since the predictions are not regarded as true and we need to be further validated using external literature support, 17 known associations are approved by the benchmark and other 7 associations are supported by the literature. The man with geriatric cancer may have prostate enlargement or prostate cancer at a higher risk while taking fluoxymesterone. On the other hand, if people has known or suspected prostate cancer, oxandrolone and drostanolone related to androgen receptors are not recommended to be taken in certain medical conditions. Previous clinical studies showed that prostate-specific antigen (PSA) plays an important tumor marker for prostate cancer in male and nandrolone phenpropionate is an androgen receptor agonist and previous study showed that it can decrease cell growth of prostate cancer LNCaP cells [46]. The primary action of cyproterone is to suppress the activity of the androgen hormones via competitive antagonism of the androgen receptor and inhibition of enzymes in the androgen biosynthesis pathway [47]. While the patients with metastatic prostate cancer were given mitomycin, the clinical results show good anti-tumour activity in metastatic prostate cancer and low toxicity in a phase II chemotherapy study [48]. Due to the androgens can stimulate the growth of prostate cancer cells, previous phase II trial study in 2012 demonstrated the hormone therapy using exemestane with or without bicalutamide may fight prostate cancer [49].

**Table 2:** Drug-prostate cancer associations

Drug ID	Drug Name	Score	Drug ID	Drug Name	Score
DB01420^a^	Testosterone Propionate	13.69	DB01216^a^	Finasteride	4.85
DB00621^b^	Oxandrolone	12.76	DB00367^a^	Levonorgestrel	4.46
DB00984^b^	Nandrolone phenpropionate	9.69	DB00262^a^	Carmustine	4.16
DB00858^b^	Drostanolone	8.14	DB06710^a^	Methyltestosterone	4.00
DB04839^b^	Cyproterone	7.78	DB00227^a^	Lovastatin	3.90
DB00687^a^	Fludrocortisone	7.44	DB00783^a^	Estradiol	3.77
DB00665^a^	Nilutamide	6.56	DB01599	Probucol	3.77
DB01185^b^	Fluoxymesterone	6.31	DB00279	Liothyronine	3.22
DB01128^a^	Bicalutamide	6.20	DB00421^a^	Spironolactone	3.01
DB01395^a^	Drospirenone	6.17	DB01406^a^	Danazol	2.99
DB00305^b^	Mitomycin	5.24	DB00624^a^	Testosterone	2.96
DB00499^a^	Flutamide	5.07	DB00396^a^	Progesterone	2.77
DB00990^b^	Exemestane	5.03	DB00928^a^	Azacitidine	2.61

a. Primary therapeutic function approved by our benchmark; b. supported by literature

#### The significant functional modules related to the drug-prostate cancer association

There are 31 genes with Z-score > 2.33 which are strongly related to 18 drugs and prostate cancer associations. We use the functional annotation analysis to investigate the functional enrichment of them via GSEA toolkits [50]. We select 4 annotation categories related to the functional pathways and processes including Biological Process (BP) in Gene Ontology (GO), KEGG, BIOCARTA, and REACTOME pathways to do the functional enrichment. The enriched functional biological processes and pathways of those genes with p-value < 0.05 are shown in Table 3 and the detailed information is obtained from GSEA and DAVID gene functional classification tool [51] see Additional file 1. The most significantly enriched terms of the regulation of apoptosis, cell cycle, p53 signaling and DNA repair indicate that those genes serve important roles in drug and prostate cancer association. The BRCA2 mutation contributing to the young-onset prostate cancer has shown to be related to the Fanconi anemia pathway and DNA repair processes [52,53]. The prostate apoptosis response (Par) factor-related proapoptotic function is associated with prostate tumor progression and sheds light on the effects of the AR pathway on cell survival and apoptosis [54]. Interestingly, this functional module may be also for programmed cell death in response to induce apoptosis in neurodegenerative disorders such as Alzheimer's and Huntington's disease pathway [55]. With literature support, Par-4 has also been initially characterized in prostate cancer and also linked with the direct induction of apoptosis and even recognized to be a new target in pancreatic cancer [56].

**Table 3:** Functional modules related to the drug-prostate cancer associations

Database	Functional modules	p-value
REACTOME	Fanconi anemia pathway	4.65E-5
KEGG	Huntington's disease pathway	1.60E-4
KEGG	p53 signaling pathway	9.72E-4
BIOCARTA	BRCA1, BRCA2 and ATR pathway	1.61E-3
GO, KEGG	Cell cycle	1.81E-3
REACTOME	DNA Repair	3.89E-3
GO	Negative regulation of cell proliferation	9.75E-3
KEGG	Alzheimer's disease pathway	1.21E-2
GO	Apoptosis	1.26E-2
KEGG	Pancreatic cancer pathway	1.69E-2
KEGG	Viral myocarditis	1.83E-2
REACTOME	Electron transport	2.13E-2

### Case study: colorectal cancer

#### Potential drug and colorectal cancer relations

We display 37 significant drug-colorectal cancer associations with Z-score > 2.33 in Table 4. There are 28 known associations between drugs and colorectal cancer approved by our benchmark and 4 associations with literature support. Pyridoxal phosphate (PLP) is an important cofactor in the reactions of amino acid metabolism and previous studies indicated that increased blood PLP levels are associated with a reduced risk of colorectal cancer [57]. The previous findings showed the association between dietary fats and colorectal cancer, and a significant inverse association was found between colorectal cancer and alpha-linoleic acid [58]. The results of this study suggest that substituting alpha-linoleic acid in the diet may reduce the risk of the colorectal cancer [58]. Previous works examined the effect of arsenic trioxide (As_2_O_3_) at various concentrations on the cell growth of the colon cancer cell lines and showed that the growth of all cell lines was gradually suppressed with As_2_O_3 _in comparison with that obtained without treatment [59,60]. Polyamines, organic compounds having two or more amino groups, have been shown to play an important role in the growth and survival in colorectal cancer [61]. We also find that spermine, a polyamine, may be a candidate target for therapeutic intervention in colorectal cancer [62]. The activity of the polyamine-synthesising enzyme, ornithine decarboxylase (ODC), is very highly expressed in proliferative HT-29 colon cancer cells comparing to those from control samples, as well as in our case study [63]. L-ornithine is apparently efficiently utilized in the ODC pathway and is also considered as growth factors involved in cell proliferation and differentiation regulated by amino acids metabolism [64]. The growing studies indicated the potential effectiveness of bortezomib in treatment of patients with HCT116 colon cancer by significantly increasing survivin expression [65,66]. The increasing evidences determined that the relationships among certain bacteria and cancer exist but the detail mechanism still unclear [67]. In our results, we interestingly find that an antibiotic drug zanamivir for bacterial infection may cause or cure colorectal cancer [68].

**Table 4:** Drug-colorectal cancer associations

Drug ID	Drug Name	Score	Drug ID	Drug Name	Score
DB0017^a^	Adenosine triphosphate	12.35	DB00313^a^	Valproic Acid	4.20
DB01593^a^	Zinc	8.66	DB00162	Vitamin A	3.77
DB00591	Fluocinolone Acetonide	8.25	DB00131	Adenosine monophosphate	3.74
DB00163^a^	Vitamin E	7.13	DB00129^b^	L-Ornithine	3.72
DB01262^a ^	Decitabine	7.02	DB00947^a^	Fulvestrant	3.46
DB00435^a^	Nitric Oxide	6.70	DB00741^a^	Hydrocortisone	3.34
DB00783^a^	Estradiol	6.40	DB01064^a^	Isoproterenol	3.21
DB00328^a^	Indomethacin	6.35	DB01128^a^	Bicalutamide	3.17
DB00755^a^	Tretinoin	6.26	DB00773^a^	Etoposide	3.15
DB00114^b^	Pyridoxal Phosphate	5.91	DB00481^a^	Raloxifene	3.10
DB00544^a^	Fluorouracil	5.62	DB00305^a^	Mitomycin	3.05
DB00132^b^	Alpha-Linolenic Acid	5.61	DB00188^b^	Bortezomib	2.77
DB00396^a^	Progesterone	5.59	DB01005^a^	Hydroxyurea	2.66
DB00997^a^	Doxorubicin	5.52	DB00619^a^	Imatinib	2.61
DB00179^a^	Masoprocol	5.09	DB02546^a^	Vorinostat	2.53
DB00558	Zanamivir	5.03	DB00928^a^	Azacitidine	2.47
DB01169^a^	Arsenic trioxide	4.38	DB00498^a^	Phenindione	2.44
DB00127^a^	Spermine	4.38	DB00548	Azelaic Acid	2.39
DB00834^a^	Mifepristone	4.21			

a. Primary therapeutic function approved by our benchmark; b. supported by literature

#### The significant functional modules related to the drug-colorectal cancer association

The enriched functional biological processes of 44 significant genes in drug-colorectal cancer associations by investigating the functional enrichment are shown in Table 5 and the detailed information see Additional file 2. The popular enriched functional terms of the gene modules are regulation of Glycogen synthase kinase 3 (GSK-3) pathway, innate immune system, Wnt signalling pathway [69] and apoptosis pathway which all appear to be promising biological processes associated with colorectal cancer. The activity of GSK3b expression in colorectal cancer patients is higher than those in their normal samples and the GSK3b inhibitor may induce apoptosis in human colorectal cancer cells [70,71]. Lipopolysaccharide (LPS) elicits several immediate proinflammatoy responses including CD14, Toll-like receptors, phosphatidylinositol-3'-kinase (PI3K)/Akt signaling, myeloid differentiation factor, nuclear factor kappa-light-chain-enhancer of activated B cells (NF-kB) transcription factors, and also promotes downstream b1 integrin function in tumor growth and progression thereby increasing the adhesiveness and metastatic capacity of colorectal cancer cells [72-74]. Here, we present evidence that S100A8/A9 actives the downstream genes associated with Mitogen-activated protein kinases (MAPK) and NF-kB signaling pathways to promote tumor growth and metastasis and the expression of S100A8/A9 on myeloid cells is also essential for development of colon tumors in mice model [75]. Untersmayr detected Fc epsilon RI (FcεRI)-positive epithelial cells in the colon cancer patients [76]. There has been a growing importance of the neurotrophin signalling in a variety of human cancers including colorectal cancer and malignant gliomas in particular [77]. Our study also denotes that the significances of p53, hypoxia inducible factor-1 alpha pathway, and vascular endothelial growth factor (VEGF) expression in colorectal cancer [78].

**Table 5:** Functional modules related to the drug-colorectal cancer associations

Database	Functional modules	p-value
BIOCARTA	GSK3 pathway	7.29E-14
KEGG	Endometrial cancer	2.30E-11
KEGG	Colorectal cancer	4.32E-9
KEGG, BIOCARTA, REACTOME	Toll like receptor pathway	5.79E-9
KEGG	Prostate cancer	1.36E-6
BIOCARTA	P53hypoxia pathway	1.93E-6
KEGG	Lung cancer	2.30E-6
KEGG	Glioma	5.82E-6
KEGG	Renal cell carcinoma	8.40E-6
KEGG	Melanoma	9.01E-6
KEGG	Chronic myeloid leukemia	1.03E-5
KEGG	VEGF signaling pathway	1.26E-5
KEGG	Innate Immune System	1.29E-5
KEGG	Fc epsilon RI signaling pathway	1.52E-5
REACTOME	Regulation of ornithine decarboxylase (ODC)	4.28E-5
KEGG	Pancreatic cancer	1.74E-4
KEGG	Wnt signaling pathway	3.34E-4
GO	Apoptosis	1.54E-3
KEGG	Neurotrophin signaling pathway	1.62E-3

## Conclusions

We integrate the information of drug, genomic and disease phenotype from available experimental data and knowledge as weighted networks and their connected relationships together. We apply disease-oriented network propagation approach for inferring and evaluating the likelihood of the probability between drugs and query disease. In our experiment, we adopt the prostate cancer and colorectal cancer as our case study and the results clearly outperform previous Cmap project. Our results are also found to be significantly enriched in both the biomedical literature and clinical trials. The success of our methods can be attributed as follows: First, we integrate heterogeneous data and knowledge about disease phenotype, chemical structure of drugs, and gene expression into our model. Second, our network propagation method combines the information not only from the single network but also derived the information from other connected homo-networks to infer the drug-disease association. Finally, our method with off-targets information gets higher performance than that with only primary drug targets in both test data. We believe that the combination of network and heterogeneous data source could help us to generate new hypotheses to infer the drug-disease associations and even speed up the drug development processes. Our study provides opportunities for future toxicogenomics and drug discovery applications but the limitation is the difficulty in distinguishing the positive and negative associations between drug and disease. In the future, we can choose different methods to calculate the chemical structural similarity between drugs, which could improve the limitations by using Tanimoto coefficient. On the other hand, our approach heavily relies on the weights for the edges in each network derived from the existing knowledge of drugs, targets, protein, disease or reported databases, or experimental results from the public database and the incompleteness of such information would limit our prediction power. We can also integrate various data sources such as drug response profile, side effect and pharmacological data and therapeutic/toxicological expression profiles to verify the reliability and confidence of the interactions.

## Competing interests

The authors declare that they have no competing interests.

## Authors' contributions

YFH carried out the design of the workflow, algorithm and molecular studies and drafted the manuscript. HYY carried out the workflow design, program development and statistical analysis. VWS participated in its overall design and coordination of the research and helped to draft the manuscript.

## Supplementary Material

Additional file 1 **The functional enrichment canonical pathways of the genes in prostate cancer**. We filter the functional enrichment canonical pathways of the overlap of the significant genes related to the drug-prostate cancer associations with at least 3 members in each functional category and p-value<0.05 using GSEA and DAVID online toolkit. Click here for file

Additional file 2 **The functional enrichment canonical pathways of genes in colorectal cancer**. We filter the functional enrichment canonical pathways of the overlap of the significant genes related to the drug-colorectal cancer associations with at least 3 members in each functional category and p-value<0.05 using GSEA and DAVID online toolkit. Click here for file

## Declarations

The publication costs for this article were funded by the corresponding author.

This article has been published as part of *BMC Medical Genomics *Volume 6 Supplement 3, 2013: Selected articles from the IEEE International Conference on Bioinformatics and Biomedicine 2012: Medical Genomics. The full contents of the supplement are available online at http://www.biomedcentral.com/bmcmedgenomics/supplements/6/S3.
